# A Comparative Analysis of the Anatomy, Phenolic Profile, and Antioxidant Capacity of *Tussilago farfara* L. Vegetative Organs

**DOI:** 10.3390/plants11131663

**Published:** 2022-06-23

**Authors:** Viviane Beatrice Bota, Andreea-Adriana Neamtu, Neli-Kinga Olah, Elisabeta Chișe, Ramona Flavia Burtescu, Flavia Roxana Pripon Furtuna, Alexandru-Sabin Nicula, Carmen Neamtu, Adrian-Marius Maghiar, Lăcrămioara-Carmen Ivănescu, Maria-Magdalena Zamfirache, Endre Mathe, Violeta Turcuș

**Affiliations:** 1 Doctoral School of Biology, Faculty of Biology, “Alexandru Ioan Cuza” University of Iași, 700505 Iași, Romania; viviane.beatrice@gmail.com (V.B.B.); ivanescu@uaic.ro (L.-C.I.); magda@uaic.ro (M.-M.Z.); 2 Faculty of Medicine, “Vasile Goldiș” Western University of Arad, 310414 Arad, Romania; aneamtu94@gmail.com (A.-A.N.); neamtu.carmen@uvvg.ro (C.N.); endre.mathe@agr.unideb.hu (E.M.); 3 National Institute for Economic Research “Costin C. Kiritescu” of the Romanian Academy, Centre for Mountain Economy (CE-MONT), 725700 Suceava, Romania; sabin.nicula@ubbcluj.ro; 4 Doctoral School of Biomedical Sciences, University of Oradea, 410081 Oradea, Romania; 5 SC PlantExtrakt SRL, Rădaia, 407059 Cluj, Romania; neliolah@yahoo.com (N.-K.O.); ramona.burtescu@plantextrakt.ro (R.F.B.); flavia@plantextrakt.ro (F.R.P.F.); 6 Faculty of Pharmacy, “Vasile Goldiș” Western University of Arad, 310414 Arad, Romania; c_elisabeta@yahoo.com; 7 Centre for Research on Settlements and Urbanism, Faculty of Geography, Babeș-Bolyai University from Cluj-Napoca, 400006 Cluj-Napoca, Romania; 8 Department of Surgical Disciplines, Faculty of Medicine and Pharmacy, University of Oradea, 410081 Oradea, Romania; amaghiar@gmail.com; 9 Institute of Nutrition, Faculty of Agricultural and Food Sciences and Environmental Management, University of Debrecen, H-4032 Debrecen, Hungary

**Keywords:** *Tussilago farfara* L., coltsfoot, morpho-anatomy, phytochemistry, polyphenol, flavonoid, antioxidant, anti-inflammatory

## Abstract

*Tussilago farfara* L., a perennial species, is a medicinal herb used in traditional medicine, mainly for the treatment of respiratory tract-related pathology. In traditional Chinese medicine, flower buds are preferred; in Europe, the leaves are used; and in some parts of India, the whole plant is utilized. This preferential usage of the plant organs might be based on differences in the chemical composition due to environmental conditions, along with preferred traditional and cultural approaches. In this article, the impact of pedoclimatic growth conditions on the morpho-anatomical development and phytochemical profile of the plant were studied on *T. farfara* in the vegetative state, collected from two different locations in the Romanian spontaneous flora, revealing significant variations. Furthermore, the antioxidant profile of the specific extracts from the aerial and subterranean plant parts is also in accordance with these discrepancies. The plant anatomy was assessed histologically by optical microscopy, while the analytical chemistry evaluation was based on LC/MS and spectral methods for the evaluation of the antioxidant and enzyme inhibitory activity. To our knowledge, this is the first comparative analysis contextually reporting on the histology, phenolic profile, antioxidant capacity, and geographical location of the vegetative form of *T. farfara*.

## 1. Introduction

*Tussilago farfara* L., popularly known as coltsfoot [1], has a longstanding medicinal reputation, rooted in its taxonomic name, which stems from the Latin words “*tussis*”, meaning “cough”, and “*ago*”, meaning “to act on” [2]. It belongs to the Asteraceae (Compositae) family, *Senecioneae* Cass. tribe. Currently, it is the only accepted species in the genus *Tussilago*, even though many similar plants have been previously assigned to it [3]. 

**Figure 1:**
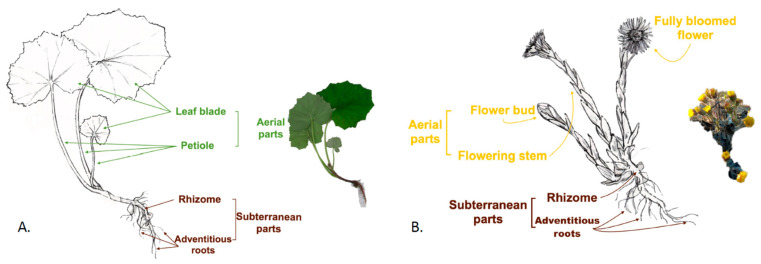
Morphology of *T. farfara* in: (**A**) vegetative form; (**B**) reproductive form. Note: original figure is not to scale.

*T. farfara* (Figure 1) is a perennial species, with a cylindrical rhizome, from which at the end of winter, scapiform flowering stems are formed bearing terminal yellow flowers [4]. After flowering, the leaves develop, forming a rosette, with a long or short petiole and a flat adaxial face. The leaf blade is almost round and deeply cordate. It is covered with hairs on both sides in its early stages, becoming glabrous on the upper side as it ages [4,5]. The plant has yellow flowers. Tubular disk flowers are centered, fertile, and have a head shaped stigma [6]. Peripheral ray flowers are multilayered, bearing a ligule with a rounded tip, a few long hairs at the base, and a bilobed stigma. Fruit are represented by cylindrical achenes of various lengths. The pappus hairs are simple, long, multilayered, and much longer than the achene [4].

*T. farfara* is native to Europe, western Asia, and northwest Africa, and has been introduced and naturalized in other parts of the world (e.g., Iceland, the United States, and Canada) [3,7]. However, besides the spontaneous flora, the species is also cultivated in countries such as Austria and Germany [8]. 

*T. farfara* is used in various cultures around the world, in both gastronomy [4,9,10] and traditional medicine. Currently, evidence-based studies on the medicinal usage of *T. farfara*, conducted both in vivo and in vitro, are emerging. To our knowledge, there is evidence for the effectiveness and potential usage of *T. farfara* extracts and isolated phytochemicals in treating diseases raging from cough, respiratory disease, lung inflammations, allergies, liver disease, cytotoxicity, cardiovascular disease, cerebral ischemia, diabetes, osteoporosis, neuropathy, neurodegenerative diseases, and gastrointestinal diseases to lung, breast, and colon cancers. The dimension of the curative potential of the plant is mostly underlaid by its antioxidant and anti-inflammatory effects. 

The chemical profiles of leaves and flower buds show a diversified content of bioactive phytochemicals, varying from sesquiterpenes, phenolic acids, flavonoids, and chromone, to pyrrolizidine alkaloids [11]. *T. farfara* is characterized by a high content of caffeic acid and its derivatives, more efficiently extracted in hydroalcoholic than aqueous preparations [12]. The predominant compounds are phenolic acids 5-caffeoylquinic acid, 3,4-dicaffeoylquinic acid, and 3,5-dicaffeoylquinic acid, due the plant’s antioxidant activity [12]. Methanolic extracts from peduncles, bracts, and phyllaries are rich in organic acids, sugars, polyols, phenols, and terpenoids [13], biochemically analogous to the metabolites found in leaves. Bearing additional glandular hairs, the leaves differ mainly in phenolic and terpenoid content [13]. The microscopic and biochemical analysis of glandular trichomes has revealed a different profile of total phenolic content, total flavonoids, and antioxidant activity between *T. farfara* populations [13,14]. The total phenolic content and antioxidant activity are proportional, according to a recent study conducted on leaf and flower extracts of *T. farfara* collected from Iran [14]. Moreover, there are significant differences in the primary and secondary metabolites composition of flower buds and fully bloomed flowers [15]. Fully open flowers have a predominantly phenylpropanoid profile, while flower buds contain mainly sugars and fatty acids [16]. The major compound identified in the essential oils from European flowers and stems was n-triclosan, followed by n-pentosan, n-hexacosane, and n-dodecane [17]. Furthermore, the flowers contained high amounts of α-fenchocamphorone [17], showing a significant difference in regards to Asian *T. farfara* essential oils. In contrast to the flowers, the leaves have been observed to contain a higher amount of phenylpropanoids, amino acids, fatty acids, and a lower amount of sugars, terpenoids, and sterols [16]. When compared with flower buds, the leaves are observed to bear higher amounts of m-cymene, 5-tridecene, cubebene, germacrene D, (-)-spathulenol, bisabolene epoxides, dibutyl phthalate, 2-hexadecanol, dehydroaromadendrene, campesterol, and stigmasterol and lower amounts of patchoulene and 17-pentatriacontene [18]. On the surface of the foliar limbs and peduncles, secretory hairs were observed, in which the presence of phenylpropanoids, polyphenols, tannins, terpenoids, lactone sesquiterpenes, and acidic and neutral lipids could be detected [19]. As research mainly focuses on flower buds, and to a lesser extent on flowers and leaves, there is little information on the content of the subterranean parts. Scarce information was found regarding the rhizome, which was only noted to contain an elevated concentration of carbohydrates (fructan, fructose) about 2 weeks after plantation, followed by a decrease [20]. 

Although *T. farfara* is a species with a long history of usage in traditional medicine [21], the safety of its therapeutic use has been at times questioned due to the presence of hepatotoxic unsaturated pyrrolizidine alkaloids (PA). However, in a review of all case studies to date of toxicity caused by orally ingested *T. farfara*, all instances were the result of the misidentification of the botanical species, and/or the attribution of PA to *T. farfara*, although they could have come from other plants established as toxic [22]. Therefore, none of the case studies evaluated is a credible source to conclude on the toxicity of the plant. However, it has been observed that the PA content can be greatly reduced in controlled cultivation settings [8].

Based on the current status of research and the interest in the chemical composition variations between the vegetative and reproductive organs [15,18,23], along with the influence exerted by the habitat on *T. farfara* development [5,10,17,19,24], in this article we aim to analyse the composition of the plant’s vegetative aerial parts, namely leaves and petioles, and the subterranean parts, namely rhizomes and adventitious roots, pointing out that the anatomy microscopically analysed in transversal sections is an element of novelty presented in this article. The samples pertain to two different locations of the Romanian spontaneous flora, which, to our knowledge, have never been the subject of a scientific publication to date. We aim to provide anatomical and analytical evidence for the differences suggested by pedoclimatic conditions between the two collection sites. The quantification of the antioxidant capacity, although thoroughly assessed in the past, is meant to anchor our results in the context of *T. farfara* studies, allowing for the expansion of the comparison between populations of the species. Moreover, the quantitative analysis of a selective phenolic profile is, to our knowledge, the most comprehensive to date reported for *T. farfara* extracts from the aerial and subterranean vegetative organs. Therefore, we hope this study will contribute to the complex assessment of the best location for sample collection, to provide an enhanced pharmacological activity for upcoming research and clinical studies using the species.

## 2. Results

### 2.1. Pedoclimatic Conditions of the Two Collection Sites of the Romanian Spontaneous Flora

**Figure 2:**
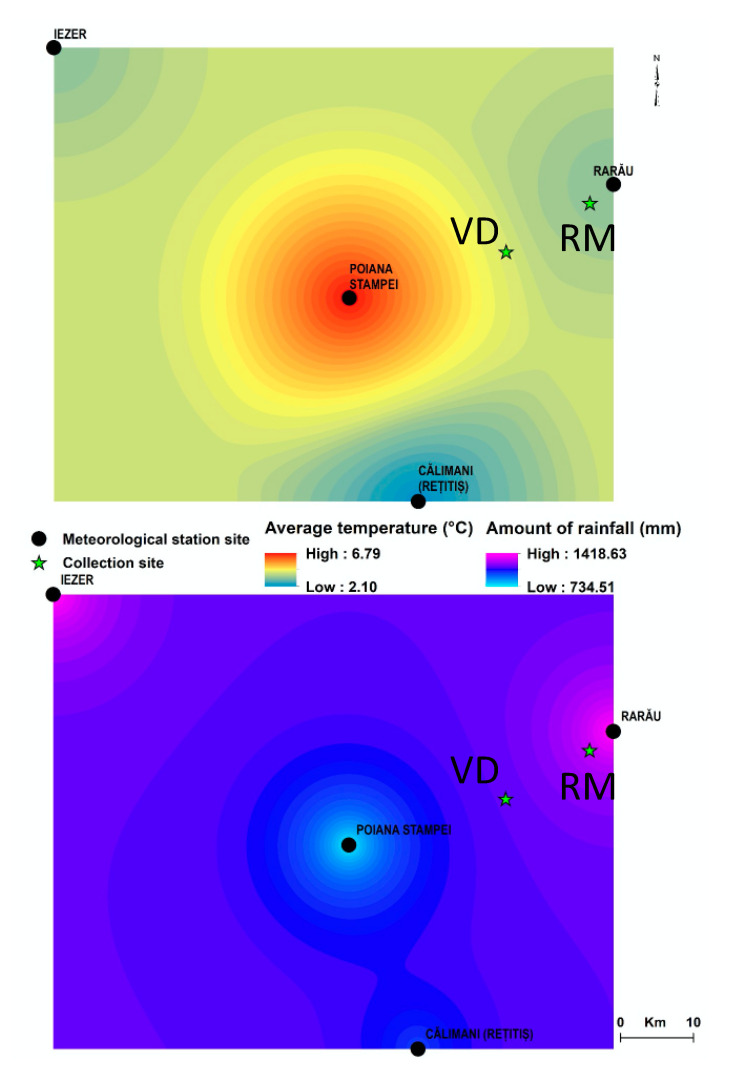
Map of average annual temperatures and average annual cumulative precipitation level for 2020 (sample collection year), for the two collection sites, based on the interpolation of data from 4 weather stations in the region (Poiana Stampei, Rarău, Călimani-Rețitiș, Iezer). The collection sites are labelled on the climatic maps as VD, for the Vatra Dornei sample, and RM, for the Rarău Mountains sample. Data source: meteomanz.com (accessed on 7 April 2022) [29].

An increasing number of studies indicate that the geographical location and the biotope could have a significant impact on the composition of plants [25,26,27]. Therefore, we have assessed the pedoclimatic conditions of the two chosen collection sites in the Romanian spontaneous flora. Both locations belong to the nemoral zone, the basement of the broad-leaved beech forests and the mixed broad-leaved and coniferous area, specific to altitudes between 600 and 1350 m [28]. The growth temperature and precipitation in the two areas were assessed (Figure 2). In Vatra Dornei (VD samples), in 2020 (the year of sample harvest) the average annual temperature was 6.8 °C, and the cumulative precipitation was reported as 732.9 mm/year. In the Rarău Mountains (RM sample), the average annual temperature in 2020 was 3.3 °C, while the cumulative precipitation was reported as 1408 mm/year.

### 2.2. Microscopic Analysis of the Anatomical Structure

**Figure 3:**
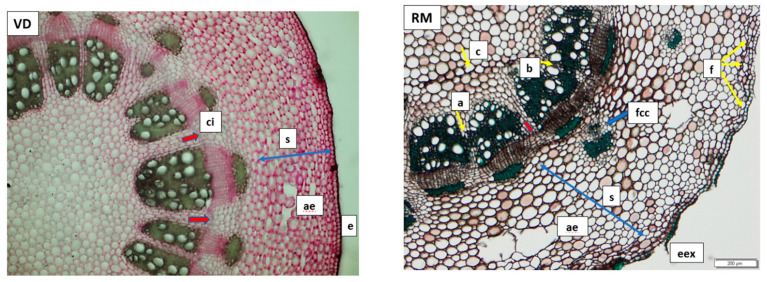
Transversal section of the *T. farfara* rhizome. Left: Vatra Dornei sample (VD); right: Rarău Mountains sample (RM). Legend: e—epidermis; eex—exfoliated epidermis; s—cortex; ae—aerenchyma; ci—interfascicular cambium; f—phellogen; fcc—cortical bundle; a—interior libriform; b—wood vessels; c—parenchymatic cells.

**Figure 4:**
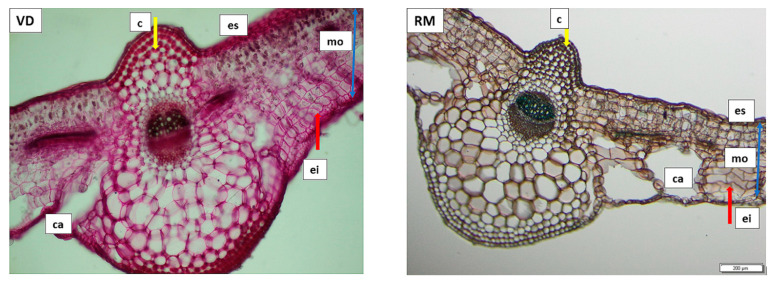
Transversal section of the *T. farfara* foliar limb through the median vein and mesophyll. Left: Vatra Dornei sample (VD); right: Rarău Mountains sample (RM). Legend: es—upper epidermis; ei—lower epidermis; mo—homogenous mesophyll; c—collenchyma; ca—air lacunae.

Representative anatomical structures of rhizomes (Figure 3) and foliar limbs (Figure 4) from the VD and RM locations were analyzed and compared below. 

Based on the rhizome microscopic analysis of the anatomical structure, exfoliation of the epidermis is only noted for the RM sample, indicating a functional interfascicular cambium (Figure 3). The latter generated, at least in some parts of the plant (denoted as “a” in Figure 3), libriform elements on the interior side, which unite the initial conductive fibers of the primary structure, or the wooden vessels (denoted as “b” in Figure 3). At the level of the cortex, the number of cell layers is similar between the samples. In the VD sample, the aerenchymae in the cortex are larger in number, but smaller in size, and situated closer to the epidermis, an aspect correlated with the low humidity conditions (as seen in Figure 2) [30,31,32]. In contrast, for the RM sample, the aerenchymae are smaller in number, located in the depth of the cortex, and have a larger diameter, due to excessive humidity (as shown in Figure 2) [30,31]. This is explained as a response to short periods of hypoxia [32]. While the number of vascular bundles is the same, the medullary rays consist of several layers of cells in the VD sample (3–6 layers) as opposed to the RM samples, which maintain the primary medullary ray structure. In the RM sample, there are 1–4 layers of medullary rays, with primary medullary rays differentiated in some places, leading to the formation of an interfascicular cambium that generates secondary liber, secondary wood, and libriform between the initial vascular bundles, uniting 2–3 vascular bundles into a larger one in certain parts of the rhizome. The discrete appearance of the interfascicular cambium (denoted by red arrows in Figure 3), indicates that the rhizome is about to shift to a secondary structure, even if the cambium is barely formed. The interfascicular cambium function and the accumulation of secondary elements is also observed by a slight compression of the parenchymal cells from the periphery of the pith, which begins to deform slightly (denoted as “c” in Figure 3) in the RM sample.

The medullary rays remain primary, as the process of differentiation occurs at the beginning. The cortical vascular bundles in the RM sample have only a few conductive elements, becoming noticeable through the presence of the periphloemic sclerenchyma sheath. It is possible, in this situation, that during ontogenesis, the pro-cambium fibers (conductive and mechanical tissues precursors) were formed outside the circle of the pro-cambium that generated the primary conducting vascular bundles. The libriform is dominant in the thickness of the wood in the RM sample, possibly explained by high humidity conditions in which the rhizome of the plant was formed. The plant avoids energy expenditure in the production of conductive vessels due to the abundant presence of water, thus compensating with libriform formation necessary for the successful mechanical support of the leaves, which are larger in size in this sample (RM).

The centric homogeneous iso-bifacial structure of the foliar limb can be observed for both the VD and RM samples (Figure 4). In both samples, there are visible air lacunae in the vicinity of the lower epidermis, which are more numerous and more clearly individualized in the RM sample. The leaf in the RM sample is characteristic of a plant grown in excessive water, also observed due to the air lacunae formed with a certain degree of symmetry, on both sides of the median vein. For the VD sample, it is observed that these air lacunae are formed by the local disorganization of cells in the lacunar parenchyma, and they differ in size, denoting an excess of humidity that appeared at some point in the life of the plant, but which is not constant. Thus, the air lacunae in the VD sample are a structural acquisition as an effect of short-term hypoxia, assuming that the sectioned leaf was, at one point, under the influence of a large amount of precipitation, leading to a closure of the ostioles of the stomata.

While both samples present with a homogeneous mesophyll, this differs between samples (Figure 4). In the VD sample, the cells are radially elongated, somewhat mimicking the palisade shape, although they remain relatively short. This aspect can be correlated with equal illumination on both sides of the leaf, yet which is insufficient to form a typical palisade tissue. The formation of air cavities substitutes for the lack of air spaces (meatuses) in between mesophilic cells, which remain relatively compact (denoted with red arrow in Figure 4). The hypodermic collenchyma island on the adaxial face of the VD sample has fewer elements than that of the RM sample. This could be possibly explained by the different size of the leaf length, as the leaf of the VD sample is smaller in size than that of the RM sample. In regards to the RM sample, the mesophyll bears tangentially flattened cells, lacking airspaces and compensating with well-defined air lacunae. The flattened shape of the cells can be correlated with the shading of the sectioned leaf, either by external objects or other leaves.

### 2.3. Liquid Chromatography Coupled with Mass Spectrometry (LC/MS) Analysis

The LC/MS analysis was carried out on two plant parts, the aerial parts (AP), namely leaves and petioles, and the subterranean parts (SP), namely rhizomes and adventitious roots, of *T. farfara*. For each location, VD and RM, the AP and SP hydro-ethanolic extracts were assessed (Figures S1–S4, found in Supplementary Materials section). Chromatograms indicate the presence of multiple polyphenols, predominantly found in the extract from leaves and petioles of the AP-VD and AP-RM samples. The 21 analyzed compounds are presented in Table 1, along with their quantitative determinations.

From 21 analyzed phytochemicals, 18 were quantified in at least one sample, while quercetin was observed to be below the detection limit in both the AP and SP samples collected from the VD site, and below the quantification limit for both RM samples; apigenin and chrysin were below the quantification limit in all 4 samples (Table 1). The main phenolic acid in *T. farfara* is chlorogenic acid, being most concentrated (2496.5 µg/g dry plant weight) in the AP-RM sample. From the class of flavonoids, hyperoside is the major compound in the AP-RM sample (701.5 µg/g dry plant weight), while myricetin is the major compound in the AP-VD sample (1042.0 µg/g dry plant weight). From the class of diterpenic phenols, carnosol could be identified in all samples in low concentrations, while carnosic acid was only quantifiable in the AP-RM sample.

The flavonoid glycosides (luteolin-7-*O*-glucoside, hyperoside, etc.) are quantifiable in the samples, while the aglycone (luteolin, quercetin) are only found in lower concentrations.

The SP-VD sample contains a noticeable amount of ellagic acid, a polyphenol from the tannins group (3021.5 µg/g dry plant weight), which cannot be detected in the remaining 3 samples. The AP samples from both locations contain gallic acid, but in much smaller amounts (48.5 and 28.5 µg/g dry plant weight in the AP-VD and AP-RM samples, respectively). 

Generally, the content in polyphenols is higher in AP samples and lower in SP samples collected from both locations, with the AP-RM sample being richer in phytochemicals than the AP-VD sample, while still maintaining a similar profile.

### 2.4. Quantitative Evaluation of Total Flavonoids and Total Phenolic Acids

The spectrophotometric quantitative determination of total flavonoids, expressed as rutoside equivalents, and total phenolic acids, expressed as caffeic acid equivalents, calculated in mg/dry plant weight, is summarized in Table 2. 

The concentration of polyphenols is higher in the AP samples as compared to the SP, with the highest amount in the AP-RM sample (Table 2). A higher concentration of flavonoids can be observed in the AP (139.35 and 383.67 mg/g dry plant weight in the AP-VD and AP-RM samples respectively), while phenolic acids are expressed at almost half of the amount (74.50 and 186.85 mg/g dry plant weight in the AP-VD and AP-RM samples, respectively). In the SP analyzed, the concentration of phenolic acids is higher in the SP-RM sample (42.50 mg/g dry plant weight), while the ratio to flavonoids remains similar to the AP-RM sample. 

### 2.5. Antioxidant Potential Analysis

The antioxidant potential of the hydro-ethanolic extracts of AP and SP samples collected from the two locations VD and RM were assessed using three established methods, namely FRAP, CUPRAC, and xanthine oxidase inhibition assay. The results are summarized in Table 3, as µM Trolox equivalents (for FRAP and CUPRAC) and µM allopurinol equivalents (for xanthine oxidase assay) per g dry plant weight. 

The results of the antioxidant capacity assessment (Table 3) follow the trends observed in polyphenol content (Table 2). The AP-RM sample, showing the highest concentration of polyphenols, also shows the highest antioxidant capacity (139 and 439.2 µM Trolox equivalents/g dry plant weight in FRAP and CUPRAC, respectively, and 70.4 nM allopurinol equivalents/g dry plant weight in the xanthine oxidase assay). The trend is confirmed by all 3 methods used (FRAP, CUPRAC, and xanthine oxidase inhibition assay), even though the quantification obtained in the xanthine oxidase inhibition assay is not significantly higher than those of the other samples. 

### 2.6. Urease, Tyrosinase, and Acetylcholinesterase Enzyme Inhibitory Capacity 

The inhibitory effect of *T. farfara* AP and SP extracts exerted on urease, tyrosinase, and acetylcholinesterase are summarized in Table 4. A strong inhibition of urease (>70%) was observed for the SP-RM sample. The AP-RM sample presented a less than 30% inhibition of the same enzyme, namely, urease. However, the AP-RM sample inhibited over 60% of the tyrosinase, and had the highest inhibitory activity against acetylcholinesterase of the 4 extracts tested (>20%). The AP-VD leaves extract inhibited more than 50% of tyrosinase, around 11% of urease, and almost 8% of acetylcholinesterase. The lowest inhibitory activity was observed for the SP-VD sample, which inhibited tyrosinase by about 28%, urease by only approximately 5%, and acetylcholinesterase at the lowest percentage of about 2%.

## 3. Discussion

Our comparative study suggests several possible correlations between the pedoclimatic conditions in which the plant has developed and the anatomy, phenolic content, and antioxidant capacity of *Tussilago farfara* L. vegetative organs.

The anatomical structure analysis of the rhizome and foliar limb of *T. farfara*, an element of novelty, reveals that the number, size, and placement of the aerenchymae might be affected by the amount of precipitation and average temperature. The most representative result was observed in the comparison of the rhizome of the plants collected from the Rarău Mountains (higher precipitation and lower temperature) where the aerenchymae were fewer, larger, and placed more in depth, while the rhizomes from Vatra Dornei, showed more numerous, smaller, and more superficially placed aerenchymae (Figure 3).

The quantitative LC/MS analysis of phenols in *T. farfara* hydro-ethanolic extracts was guided by previous publications suggesting the phytochemicals found in the species. One of the first studies to report the presence of three phenolic acids (*p*-hydroxybenzoic, *cis*-, *trans*-*p*-coumaric, and *cis*-, *trans*- chlorogenic) in leaves of *T. farfara* populations from Poland, along with six flavonoids, identified as kaempferol and quercetin and their derivates, respectively, dates back to 2013 [11]. While confirming the presence of the previously mentioned phytochemicals, both the extracts from the Romanian spontaneous flora are more concentrated in chlorogenic acid, while the aerial parts of the Rarău Mountains sample also contains a higher amount of isoquercitrin per gram dry plant weight (Table 1) following our extraction protocol. The presence of chlorogenic acid and flavonoids, such as isoquercitrin, rutin, quercitrin, quercetin, and kaempferol, was confirmed using HPLC-MS for *T. farfara* leaves in samples from Romania [33]. The presence of gallic acid, ferulic acid, caffeic acid, kaempferol, quercetin, and *p*-hydroxybenzoic acid was reported in the stems and flowers, with apigenin and luteolin below the detection limit for samples from Slovakia [34], similar to the vegetative organs of Romanian origin, where only luteolin could be quantified in the aerial parts (leaves and petioles) of the Rarău Mountains sample (Table 1). Recently, ferulic acid was detected in *T. farfara* leaves of Bulgarian origin, along with a much higher amount of caffeic acid [24]. Our analysis revealed the presence of multiple phenols in the two Romanian samples, found to be most concentrated in the aerial parts extract of the Rarău Mountains sample, which, in correlation with the pedoclimatic conditions, could suggest the beneficial effect on the phytochemical composition of the plant at an increased cumulative precipitation level and a decreased annual temperature (Figure 2). Furthermore, the subterranean parts (rhizome and adventitious roots) of the Romanian *T. farfara* collected contain the same types of phenolic acids observed in the aerial parts (leaves and petioles), but in reduced quantity (Table 1). Similarly, flavonoids are present in lesser amounts at this level (Table 1), which is devoid of light.

Our study has identified and quantified more phenolic acids and flavonoids than have ever before been identified in the species, yielding the most complex phenolic profile of *T. farfara* to date. To our knowledge, this is the first time that salicylic acid and carnosol have been reported in the *T. farfara* plant, and luteolin, myricetin, naringenin, and carnosic acid in the vegetative aerial parts (leaves and petioles). As different parts of the plant were considered in previously published works, it is difficult to put the quantitative results into a reliable comparative framework. However, the trend observed in the quantification of the individual compounds is followed in the assessment of the total flavonoids and total phenolic acids, confirming higher concentrations in the aerial parts extracts, with the most concentrated sample being found in the aerial parts of the Rarău Mountains collection sample.

Furthermore, the trend observed in the LC/MS and spectrophotometric quantitative analysis is consistent with the highest antioxidant capacity, observed for the extract of the aerial parts sample collected from the Rarău Mountains, in all 3 methods—FRAP, CUPRAC, and xanthine oxidase inhibition assay. Next comes the extract of the vegetative aerial parts collected from Vatra Dornei, bearing about half the antioxidant capacity of the previous extract using the FRAP method, less than half for the CUPRAC method, and a less significant difference using the xanthine oxidase assay. Finally, the subterranean parts sample collected from the Rarău Mountains extract is classified, at around half the antioxidant capacity of aerial parts collected from the Vatra Dornei extract, in both the FRAP and CUPRAC methods. The lowest antioxidant capacity was observed for the subterranean parts sample collected from the Vatra Dornei extract, confirmed by all 3 methods used. In the literature, no quantification was found to be directly comparable through association of methodology and plant parts analyzed; however, the qualitative comparison of leaves (higher antioxidant capacity) and roots (lower antioxidant capacity) aligned our research with previous reports (summarized in the Supplementary Materials Section in Table S1) [12,14,35,36,37,38,39,40,41,42,43,44,45,46,47,48,49,50].

The elevated antioxidant capacity and vast phenolic content of the *T. farfara* extracts make the samples from the Romanian spontaneous flora worth further exploration in the field of pharmacologic research beyond the traditional usage as a treatment for respiratory ailments. Currently, there is notable focus of original research on the potential usage of the plant and its constituents in cancerous pathologies, namely lung, colorectal, breast, and liver cancer, as an adjuvant for standard therapy, due to its synergistic effect on various pathways involved in tumor development and its ability to aid in the reduction of the side effects of standard of care. For example, in lung cancer, isolated *T. farfara* polysaccharides appear to act as an immunomodulator by reducing the expression of CD279 and CD274 in peripheral blood and tumor tissue lymphocytes [51], increasing the efficiency of conventional antitumor therapy and reducing neutropenia, with a comparable effect to recombinant CSF Neupogen [52]. They also appear to promote reparative regeneration of the intestinal epithelium damaged by polychemotherapy [53,54]. In colorectal cancer, they show an antitumorigenic and anti-inflammatory effect via NF-κB, Nrf2, Wnt, and β-catenin pathways modulation [55,56,57] and induce cytotoxicity in HT-29 colorectal cancer cells in a dose-dependent manner [58]. In breast cancer, isolated *T. farfara* sesquiterpenoids exert an antiproliferative activity by interacting with cysteine residues of proteins [59]. In hepatic carcinoma, the methanolic extract of leaves induces apoptosis in Huh7 cells by activating the TRAIL pathway, inhibiting MKK7-TIPRL interaction, and increasing MKK7/JNK phosphorylation [60]. In correlation with our analysis revealing concentrations up to 2.5 mg/g dry plant weight of chlorogenic acid, the antitumorigenic activities of the extract could be attributed to the synergistic effect of all phytochemicals contained, aiding the well-studied effect of the active principle. Chlorogenic acid has been reported: (1) to modulate the NF-κB signaling pathway in breast cancer models [61]; (2) to induce S-phase cell-cycle arrest and apoptosis in colorectal cancer models [62]; (3) to inhibit proliferation, invasion, and metastasis by downregulating DNMT1 protein expression in hepatic carcinoma models [63]; (4) and to inhibit cellular proliferation by targeting annexin A2 in lung cancer models [64], as an isolated phytonutrient.

Furthermore, in the enzyme inhibition assays, the extract of the subterranean parts from the Rarău Mountains collection sample strongly inhibited urease (by over 70%). The enzyme is specific to some bacteria, determining their virulence [65], so its inhibition could indicate a strong antimicrobial effect of the extract. This was also postulated as a therapeutic potential for *T. farfara* [34,45,66,67,68,69,70,71,72]. 

The strong inhibitory potential of the aerial parts extract from the Rarău Mountains against tyrosinase could be possibly attributed to its high concentration of polyphenols [73]. Tyrosinase is an enzyme involved in the biosynthesis of melanin and neuromelanin, as oxidative products downstream from L-DOPA [74]. DOPA deficiency is correlated with Parkinson’s disease [75], so we can suggest that the analyzed hydro-ethanolic extracts from *T. farfara* could also have neuroprotective capacity, validating claims of previous studies [36,43,44,76,77,78]. 

Acetylcholinesterase, an enzyme involved in the conversion of acetylcholine, a neurotransmitter whose deficiency is a major cause of Alzheimer’s disease [79], is weakly inhibited by the analysed extracts, with the highest inhibition obtained for the aerial parts extract from the Rarău Mountains. The inhibitory potential on acetylcholinesterase of *T. farfara* leaf extracts was previously reported in the analysis of an ethyl acetate extract, along with an HPLC analysis revealing a high content of phenolic compounds, such as chlorogenic acid [45]. Our results do could not confirm this previous claim, and neither has our literature analysis yielded any results in the case of Alzheimer’s disease; as a therapeutic effect on this disease, *T. farfara* has never previously, to our knowledge, been the subject of an original research article. 

To conclude, our analysis of *T. farfara* from two different locations in the Romanian spontaneous flora has revealed significant differences at the anatomical and chemical level. The species is worth exploring further, due to its biological activities, exhibited by its phytochemical composition and synergistic beneficial effects in the modulation of a multitude of human pathophysiologic pathways. With this scope in mind, we have designed our study to be used as a guide regarding the plant characteristics for *T. farfara* from Vatra Dornei and the Rarău Mountains (Romania) and to give notice regarding the discrepancies in the content according to geographical location specificities of plant material used for various biological and medicinal applications.

## 4. Materials and Methods

### 4.1. Vegetal Material Collection

The plant material is represented by the aerial parts (AP), namely leaves and petioles, and the subterranean parts (SP), namely rhizomes and adventitious roots, of *T. farfara*, collected on 19 June 2020 (in the afternoon), for both samples, from the spontaneous flora of Romania, in the following locations:

- Vatra Dornei (Dorna-Arini), Romania; 1027 m altitude; GPS coordinates N 47°22′25.507″ E 25°27′59.904″—denoted in text as VD samples—registered with the voucher number 1251 (Figure S5 Supplementary Materials) in the Herbarium of the Plant Systematics discipline, part of the Department of Biology and Life Sciences of the Vasile Goldiș Western University of Arad (Romania);
- Rarău Mountains, Romania; 1244 m altitude; GPS coordinates N 47°26′7.318″ E 25°34′21.802″—denoted in text as RM samples—registered with the voucher number 1252 (Figure S6 Supplementary Materials) in the Herbarium of the Plant Systematics discipline, part of the Department of Biology and Life Sciences of the Vasile Goldiș Western University of Arad (Romania).

### 4.2. Microscopic Analysis of the Anatomical Structure

For the analysis of the internal anatomical structure of the main vegetative organs, transversal sections were performed through the rhizome and foliar limb, using a botanical razor and a hand microtome, and following the protocol of Șerbănescu-Jitariu et al. (1983) [80]. The samples were treated with sodium hypochlorite for 5 min, then washed twice with acetic acid solution 1.5%. The sections were subjected to a double-coloring process. Firstly, samples were incubated with iodine green for 10 s, then washed twice with 90% ethanol and purified water. Secondly, samples were incubated with ruthenium red for 1 min, then washed twice with purified water. Microscopy was performed using a Novex Holland microscope, with incorporated camera, operated with the BEL Capture Software, and an Olympus BX43 microscope with Olympus XC30 camera, operated with cellSens Life Science Imaging Software, Olympus, respectively.

### 4.3. Preparation of Hydroalcoholic Extracts

Hydroalcoholic extracts of *T. farfara* were prepared after drying the vegetal product at room temperature, protected from light, for 7 days. The aerial (leaf and petioles) and subterranean (rhizome and adventitious roots) parts were extracted in 70% ethanol, in a 1:5 ratio (mass/volume) of shredded plant to solvent [81,82]. The extracts were obtained after 10 days of maceration at room temperature, protected from light, complemented with 20 min/day stirring at 700–900 rpm, followed by vacuum filtration through analytical filter paper, type W, for qualitative determinations. The filtrate was calibrated at the initial volume for all extracts. The 70% vol. ethanol was prepared using 96% ethanol purchased from Coman Prod, Ilfov, Romania and purified water produced by Millipore. The final concentration was 0.2 g dry plant weight/mL extract. 

### 4.4. LC/MS Analysis

The LC/MS analysis was performed on a Shimadzu Nexera I LC/MS—8045 (Kyoto, Japan) UHPLC system equipped with a quaternary pump and autosampler, and an ESI probe and quadrupole rod mass spectrometer, respectively. The separation was carried out on a Luna C18 reversed-phase column (150 mm × 4.6 mm × 3 mm, 100 Å), Phenomenex (Torrance, CA, USA). The column was maintained at 40 °C during the analysis. 

The mobile phase was a gradient made from LC/MS grade methanol (Merck, Darmstadt, Germany) and ultra-purified water prepared using a Simplicity Ultra Pure Water Purification System (Merck Millipore, Billerica, MA, USA). LC/MS grade formic acid (Merck, Darmstadt, Germany) was used as an organic modifier at a concentration of 2%. The flow rate used throughout the analysis was 0.5 mL/min, for a total time of 35 min. The gradient was set as follows: 5-to-15% methanol increased linearly in 3 min, maintained constant for 3 min, increased linearly 5-to-21% in 3 min, maintained for 4 min, increased linearly 1-to-30% in 5 min, maintained at 30% for 4 min, increased linearly 30-to-50% in 4 min, maintained at 50% for 3 min, and decreased to 5% until the end of the analysis.

The detection was performed on a quadrupole rod mass spectrometer operated with electrospray ionization (ESI), both in negative and positive multiple reaction monitoring (MRM) ion mode (see Table S2). The interface temperature was set at 300 °C for vaporization and as drying gas, nitrogen was used at 35 psi, 10 L/min, respectively. The capillary potential was set to +3000 V.

As standards, caffeic acid, *trans-p*-coumaric acid, salicylic acid, chlorogenic acid, apigenin, chrysin, luteolin, luteolin-7-*O*-glucoside, quercetin, rutoside (rutin), naringenin, carnosic acid, ellagic acid, carnosol, kaempferol, myricetin, hyperoside, isoquercitrin, ferulic acid, and gallic acid were used (Table S3). Standards at various concentrations were injected at a volume of 1 µL for identifications and calibration curves for quantification.

### 4.5. Quantitative Evaluation of Total Flavonoids 

The determination of total flavonoid concentration was carried out spectrophotometrically and expressed in rutoside, according to the method of the Romanian Pharmacopoeia, ed. X [83]. For the determinations, a Cintra 101, GBC Australia, double-fascicle spectrophotometer, and special glass cuvettes with a 10 mm optical path were used. The monochromator slit was 1.5 nm. The determination was carried out at 430 nm. From each extract, we prepared 3 samples, and each sample was read 3 times. The results represent the mean of the 3 independent determinations; the relative standard deviation was calculated as well.

Samples were prepared from 0.5 mL extract, to which we added 5 mL sodium acetate 10%, and 3 mL aluminium chloride 2.5%; the mixture was brought to 25 mL with methanol. Blind samples were prepared using 0.5 mL extract (equivalent of 0.1 g), and 8 mL purified water, brought to 25 mL with methanol. The reading of the samples was performed after 15 min of rest, standard sample versus blind sample. 

Quantitative determination was carried out by measuring solutions containing 4.08, 8.16, 12.24, 16.32, and 20.40 mg/mL rutoside, respectively, under similar conditions. An absorbance calibration curve was constructed as a function of concentration and by interpolation of the absorbances of the samples, i.e., based on the calibration curve equation, and taking into account the sample dilution, we calculated the total flavonoid content of the extracts. The data for all spectral calibration curves are presented in Table S4.

### 4.6. Quantitative Evaluation of Total Phenolic Acids

The determination of the concentration of total phenolic acids was carried out spectrophotometrically and expressed as caffeic acid equivalents, according to the method of the Romanian Pharmacopoeia, ed. IX [84].

For the determinations, Cintra 101, GBC Australia, double-phase spectrophotometer, and special glass cuvettes with an optical path of 10 mm were used. The monochromator slit was 1.5 nm. The determination was carried out at 715 nm. From each extract, we prepared 3 samples and read each sample 3 times. The results represent the mean of the 3 determinations; the relative standard deviation was calculated as well.

Samples were prepared from 1 mL extract (equivalent of 0.2 g dry plant weight), to which we added 0.5 mL phospho-wolfram reagent; the mixture was brought to 25 mL with 15% sodium carbonate solution. Blind samples were prepared using 1 mL extract, made up to 25 mL with 15% sodium carbonate solution. The reading of the samples was performed after 2 min of rest, sample versus blind sample. 

Quantitative determination was carried out by measuring solutions containing 4.2, 8.4, 12.6, 16.8, and 21 mg/mL of caffeic acid, respectively, under similar conditions. An absorbance calibration curve was constructed as a function of concentration and by interpolation of the absorbances of the samples, i.e., based on the calibration curve equation, and taking into account the sample dilution, we calculated the content of total phenolic acids in the extracts.

### 4.7. Antioxidant Potential Analysis

#### 4.7.1. FRAP Assay

The FRAP method is based on the evaluation of the possibility of inhibition by antioxidants of iron oxidation from Fe(II) to Fe(III). 

The FRAP reagent was prepared by adding 2.5 mL 20 mM ferric chloride and 25 mL acetate buffer pH 3.6 to 2.5 mL 10 mM TPTZ solution in 40 mM hydrochloric acid. Samples were prepared by adding 0.8 mL solution containing 1 µL extract (AP-VD, AP-RM, and SP-RM), respectively, to 6 mL FRAP reagent 5 µL for SP-VD. The blank was obtained from a 6 mL FRAP reagent and 0.8 mL purified water. The standard was prepared from a 6 mL FRAP reagent, to which we added 0.8 mL solution containing 10, 20, 30, and 40 µg Trolox, respectively.

The determinations were carried out at a wavelength of 593 nm. The antioxidant capacity was expressed as mM Trolox equivalent/g dry plant weight, using the calibration curve obtained for the standard [85,86].

#### 4.7.2. CUPRAC Assay

The CUPRAC method evaluates the potential of antioxidants to prevent copper oxidation from Cu(I) to Cu(II).

The CUPRAC reagent is obtained by adding 1 mL 10 mM copper chloride solution and 1 mL ammonium acetate buffer pH 6.8 to 1 mL 7.5 mM neocuproine solution. The samples were obtained as follows: to 3 mL CUPRAC reagent, we added 1.1 mL solution containing 12 µL extract (equivalent of 2.4 µg dry plant weight, AP-VD, AP-RM, and SP-RM), or 6 µL of SP-VD extract. The blank was prepared from 3 mL CUPRAC reagent and 1.1 mL purified water. Mixtures of 3 mL CUPRAC reagent and 1.1 mL solution containing 11.4, 22.8, 34.2, and 45.6 µg Trolox respectively were used as standards.

Determinations were made at a wavelength of 450 nm. The antioxidant capacity was expressed as mM Trolox equivalent/g dry plant weight, using the calibration curve obtained from the standard [87].

#### 4.7.3. Xanthine-Oxidase Inhibition Assay

Xanthine oxidase is an oxidative enzyme in the body whose expression increases with increased oxidative stress. Using xanthine as a substrate, we evaluated the possibility of inhibiting xanthine oxidase activity in the presence of the extracts studied.

Samples were obtained by adding to each 2 µL sample solution, 3.9 mL phosphate buffer at pH = 7.4, respectively, 0.6 mL xanthine oxidase 0.2 U/mL. All mixtures were incubated at 25 °C for 10 min. Then, 4.5 mL of 0.15 mM xanthine was added per sample and incubated at 25 °C for 30 min. The negative control was prepared from 1.5 mL ultra-purified water, to which 3.9 mL phosphate buffer pH = 7.4 were added, respectively, to 0.6 mL xanthine oxidase 0.2 U/mL. The mixture was incubated at 25 °C for 10 min. Then, 4.5 mL of 0.15 mM xanthine were added, and the mixture was further incubated at 25 °C for 30 min. As a blank, 9 mL ultra-purified water were used, to which 3.9 mL phosphate buffer pH = 7.4, and 0.6 mL xanthine oxidase 0.2 U/mL were added, respectively. The mixture was incubated at 25 °C for 40 min. As standard, a solution of 1.5 mL ultra-purified water containing 195 µg allopurinol to which 3.9 mL phosphate buffer pH = 7.4 were added, and 0.6 mL xanthine oxidase 0.2 U/mL. The mixture was incubated at 25 °C for 10 min, then 4.5 mL of 0.15 mM xanthine was added and incubated further at 25 °C for 30 min.

The determination was performed at 293 nm wavelength. Antioxidant capacity is expressed as the percentage of inhibition, and in nM allopurinol equivalent/g dry plant weight, respectively [88].

### 4.8. Enzyme Inhibition Assays

#### 4.8.1. Urease Enzyme Inhibition Assay

Samples for anti-urease activity were prepared by adding 1 µL extract (AP-VD, AP-RM, and SP-RM), respectively, 5 µL for SP-VD, to 0.2 mL urease 0.1 mg/mL, 4 mL Tris-hydrochloric acid buffer at pH = 8, and 0.2 mL urea 60 mM, then the mixtures were incubated at 30 °C for 20 min. Finally, 2 mL of 10% trichloroacetic acid and 0.5 mL Nessler reagent were added. For the negative control sample, 0.2 mL ultra-purified water, 0.2 mL urease 0.1 mg/mL, 4 mL Tris-hydrochloric acid buffer pH = 8, and 0.2 mL urea 60 mM were used, and the mixture was incubated at 30 °C for 20 min. Finally, 2 mL of 10% trichloroacetic acid and 0.5 mL Nessler reagent were added. As a blank, ultrapure water was used, while as a positive control, 0.181 mg thiourea was added. 

The determination was carried out at wavelength of 436 nm and the anti-urease capacity was expressed as the percentage of inhibition [89].

#### 4.8.2. Tyrosinase Enzyme Inhibition Assay

For tyrosinase inhibition activity, 0.4 mL tyrosinase 250 U/mL and 7.4 mL phosphate buffer pH = 7.4 to 0.05 mL of extract were added, then the mixtures were incubated at 30 °C for 15 min. Finally, 0.2 mL L-DOPA 10 mM were added. The negative control sample was prepared as follows: to 0.4 mL tyrosinase 250 U/mL, we added 7.8 mL phosphate buffer pH = 7.4, then incubated the mixture at 30 °C for 15 min. Finally, 0.2 mL L-DOPA 10 mM were added. The blank used was ultra-purified water and the positive control was 0.34 mg ascorbic acid.

The wavelength used for determination was 475 nm, and the anti-tyrosinase capacity was expressed as the percentage of inhibition [90].

#### 4.8.3. Acetylcholinesterase Enzyme Inhibition Assay

To evaluate the anti-acetylcholinesterase activity, 6 mL of Tris-hydrochloric acid buffer, 50 µL acetylcholinesterase 6 U/mL, was added to 20 µL extract (AP-VD, AP-RM, and SP-RM), respectively, to 10 µL SP-VD extract in 1 mL purified water, then the mixtures were incubated at 30 °C for 15 min. Finally, we added 0.1 mL of 3 mM 5,5′-dithiobis-2-nitrobenzoic acid and 100 µL 15 mM acetylthiocholine iodide. The negative control sample was obtained by adding to 1 mL purified water, 6 mL Tris-hydrochloric acid buffer, and 50 µL acetylcholinesterase 6 U/mL; then the mixtures were incubated at 30 °C for 15 min. Finally, we added 100 µL of 3 mM 5,5′-dithiobis-2-nitrobenzoic acid and 100 µL of 15 mM acetylthiocholine iodide. The blank used was ultra-purified water and the positive control was 2.18 mg galantamine. 

Determinations were performed at a 405 nm wavelength. The inhibition capacity of acetylcholinesterase was expressed as the percentage of inhibition [90].

### 4.9. Statistical Analysis

The statistical analysis was carried out with Dixon’s Q test. Each compound was compared between samples, using the 95% interval of confidence. The test was carried out in accordance with the protocols previously described [91,92].

## 5. Conclusions

Our experimental analysis of *T. farfara* aerial (namely, leaves and petioles) and subterranean (namely, rhizome, and adventitious roots) parts, comparing the morpho-anatomical, ecological, and phytochemical aspects of plants pertaining to two collection sites of the Romanian spontaneous flora (namely, Vatra Dornei and the Rarău Mountains), has revealed the following: (1) The presence of multiple polyphenols, found in the highest concentration in the aerial parts extract of the Rarău Mountains sample, in correlation with the pedoclimatic conditions, could suggest the beneficial effect of an increased cumulative precipitation level and decreased annual temperature on the phytochemical composition of the plant; (2) The antioxidant profile of the aerial and subterranean parts extracts is also in accordance with these discrepancies, and shows the highest antioxidant capacity for the aerial parts from the Rarău Mountains, using all 3 assessment methods—FRAP, CUPRAC, and xanthine oxidase inhibition assay.

## Figures and Tables

**Table 1:** Quantitative determination of polyphenols by LC/MS in the hydro-ethanolic extract of the aerial and subterranean parts of *T. farfara*, in accordance with the standard calibration curves for purified references.

Sample Compounds	VD Sample [µg/g Dry Plant Weight]		RM Sample [µg/g dry Plant Weight]	
	AP	SP	AP	SP
Caffeic acid	91.5 ± 0.8	62.5 ± 0.5	291.0 * ± 1.9	40.5 ± 0.3
Chlorogenic acid	1097.0 ± 7.9	72.0 ± 0.4	2496.5 * ± 12.7	576.5 ± 4.9
*Trans-p*-coumaric acid	43.5 ± 0.3	<QL	10.5 ± 0.1	<DL
Ferulic acid	<DL	<DL	60.5 ± 0.4	<DL
Gallic acid	48.5 ± 0.4	<DL	28.5 ± 0.2	<DL
Ellagic acid	<DL	3021.5 ± 25.5	<DL	<DL
Salicylic acid	45.5 ± 0.4	40.5 ± 0.3	48.5 ± 0.3	64.0 ± 0.5
Luteolin	<QL	<QL	13.0 ± 0.1	<QL
Luteolin-7-*O*-glucoside	3.5 ± 0.1	3.5 ± 0.1	4.0 ± 0.1	3.5 ± 0.1
Quercetin	<DL	<DL	<QL	<DL
Hyperoside	7.0 ± 0.1	8.0 ± 0.2	701.5 * ± 4.9	7.0 ± 0.1
Rutoside	<QL	<QL	17.5 ± 0.1	<QL
Isoquercitrin	<DL	<DL	529.5 ± 4.3	<DL
Kaempferol	35.5 ± 0.2	11.0 ± 0.1	269.5 * ± 1.5	<DL
Myricetin	1042.0 ± 8.8	<DL	<DL	<DL
Apigenin	<QL	<QL	<QL	<QL
Chrysin	<QL	<QL	<QL	<QL
Naringenin	<QL	<QL	4.0 ± 0.1	<QL
Carnosol	1.5 ± 0.1	1.5 ± 0.1	1.5 ± 0.1	1.5 ± 0.1
Carnosic acid	<DL	<DL	50.5 ± 0.3	<DL

Abbreviations: VD sample—Vatra Dornei sample collection location; RM sample—Rarău Mountains sample collection location; AP—aerial parts extract; SP—subterranean parts extract; <QL—compound below quantification limit; <DL—compound below detection limit/absence of the compound. Note: Values represent the mean ± standard deviations of three independent measurements. Statistical analysis was carried out with Dixon’s Q test; * represents *p* < 0.05 of the sample compared to the same compound in the other samples.

**Table 2:** Quantity of total flavonoids and total phenolic acids in aerial (AP) and subterranean parts (SP) of *T. farfara* hydro-ethanolic extracts.

Sample	Total Flavonoids Expressed as Rutoside	Total Phenolic Acids Expressed as Caffeic Acid
	Average Concentration [mg/g Dry Plant Weight]	Average Concentration [mg/g Dry Plant Weight]
**AP-VD**	139.35 * ± 1.75	74.50 * ± 0.87
**AP-RM**	383.67 * ± 3.58	186.85 * ± 2.08
**SP-VD**	29.75 ± 0.31	22.10 ± 0.28
**SP-RM**	20.15 * ± 0.19	42.50 * ± 0.42

Abbreviations: VD sample—Vatra Dornei sample collection location; RM sample—Rarău Mountains sample collection location; AP—aerial parts extract; SP—subterranean parts extract; <QL—compound below quantification limit; <DL—compound below detection limit/absence of the compound. Note: Values represent the mean ± standard deviations of three independent measurements. Statistical analysis was carried out with Dixon’s Q test; * represents *p* < 0.05 of the sample compared to the same compound in the other samples.

**Table 3:** Antioxidant capacity of aerial (AP) and subterranean parts (SP) of *T. farfara* hydro-ethanolic extracts.

Sample	FRAP [µM Trolox Equivalent/g Dry Plant Weight]	CUPRAC [µM Trolox Equivalent/g Dry Plant Weight]	Xanthine Oxidase [nM Allopurinol Equivalent/g Dry Plant Weight]
**AP-VD**	55.1 ± 0.5	144.3 ± 1.2	58.2 ± 0.6
**AP-RM**	139.7 * ± 1.2	439.2 * ± 3.8	70.4 ± 0.6
**SP-VD**	18.0 ± 0.1	55.9 ± 0.5	56.0 ± 0.4
**SP-RM**	30.0 ± 0.3	94.7 ± 1.0	66.6 ± 0.5

Abbreviations: VD sample—Vatra Dornei sample collection location; RM sample—Rarău Mountains sample collection location; AP—aerial parts extract; SP—subterranean parts extract. Note: Values represent the mean ± standard deviations of three independent measurements. Statistical analysis carried out with Dixon’s Q test; * represents *p* < 0.05 of the sample compared to the same compound in the other samples.

**Table 4:** The inhibitory capacity of aerial (AP) and subterranean parts (SP) of *T. farfara* hydro-ethanolic extracts on the enzymes urease, tyrosinase, and acetylcholinesterase.

Sample	Urease [% Inhibition]	Tyrosinase [% Inhibition]	Acetylcholinesterase [% Inhibition]
**AP-VD**	11.43 ± 0.08	55.36 ± 0.10	7.95 ± 0.01
**AP-RM**	27.67 ± 0.10	63.35 ± 0.09	20.59 * ± 0.03
**SP-VD**	5.68 ± 0.02	28.18 * ± 0.06	2.41 ± 0.01
**SP-RM**	70.61 * ± 0.11	49.72 ± 0.09	9.19 ± 0.01

Abbreviations: VD sample—Vatra Dornei sample collection location; RM sample—Rarău Mountains sample collection location; AP—aerial parts extract; SP—subterranean parts extract. Note: (1) Values represent the mean ± standard deviations of three independent measurements. Statistical analysis carried out with Dixon’s Q test; * represents *p* < 0.05 of the sample compared to the same compound in the other samples. (2) As positive controls, for the urease assay—thiourea 0.181 mg yielded an inhibition of 61.67%, for the tyrosinase assay—ascorbic acid 0.34 mg yielded an inhibition of 96.53%, while for the acetylcholinesterase assay—galantamine 2.18 mg yielded an inhibition of 31.66%.

## Data Availability

The data presented in this study are available within the article and/or supplementary materials.

## Supplementary Materials

The following supporting information can be downloaded at: https://www.mdpi.com/article/10.3390/plants11131663/s1, Figure S1. LC/MS chromatogram of the hydroalcoholic extract obtained from leaves of *T. farfara* L. from the Rarău Mountains (RM) compounds analyzed; Figure S2. LC/MS chromatogram of the hydroalcoholic extract obtained from roots of *T. farfara* L. from the Rarău Mountains (RM); Figure S3. LC/MS chromatogram of the hydroalcoholic extract obtained from leaves of *T. farfara* L. from Vatra Dornei (VD); Figure S4. LC/MS chromatogram of the hydroalcoholic extract obtained from roots of *T. farfara* L. from Vatra Dornei (VD); Figure S5. *Tussilago farfara* L. collected from Vatra Dornei, registered with the voucher number 1251 in the Herbarium of the Plant Systematics discipline, part of the Department of Biology and Life Sciences of the Vasile Goldiș Western University of Arad (Romania); Figure S6. *Tussilago farfara* L. collected from the Rarău Mountains, registered with the voucher number 1252 in the Herbarium of the Plant Systematics discipline, part of the Department of Biology and Life Sciences of the Vasile Goldiș Western University of Arad (Romania); Table S1. Antioxidant activity of *T. farfara* extracts and isolated phytochemicals. Summarized results of original articles sorted by the plant part responsible and the extraction method; Table S2. The standards for chromatographic and spectral data; Table S3. The standards used for LC/MS analyses, and Table S4. The spectral calibration curves data.
