# Supplementary material for: A Comparative Analysis of the Anatomy, Phenolic Profile, and Antioxidant Capacity of Tussilago farfara L. Vegetative Organs
Source: Plants (Basel). 2022 Jun 23;11(13):1663. doi: 10.3390/plants11131663 (PMC9269468; doi:10.3390/plants11131663)
Supplement: Supplementary file 1 [file plants-11-01663-s001.zip › plants-1758064-supplementary.pdf]

Supplementary Materials

A comparative analysis of the anatomy, phenolic profile, and antioxidant capacity of *Tussilago farfara* L. vegetative organs

Viviane-Beatrice Bota [1,2,3,†], Andreea-Adriana Neamtu[2,4,†], Neli-Kinga Olah [5,6], Elisabeta Chișe[6], Ramona-Flavia Burtescu[5], Flavia-Roxana Pripon Furtuna[5], Alexandru-Sabin Nicula[3,7], Carmen Neamțu[2], Adrian-Marius Maghiar[8], Lăcrămioara-Carmen Ivănescu[1], Maria-Magdalena Zamfirache[1], Endre Mathe[2,9], and Violeta Turcuș[2,3,*]

1 Doctoral School of Biology, Faculty of Biology, "Alexandru Ioan Cuza" University of Iași, 700505 Iași, Romania; V.B.B. viviane.beatrice@gmail.com; L.C.I. ivanescu@uaic.ro; M.M.Z. magda@uaic.ro,

2 Faculty of Medicine and Life Sciences, "Vasile Goldiș" Western University of Arad, 310414 Arad, Romania; V.B.B., A.A.N. aneamtu94@gmail.com, C.N. neamtu.carmen@uvvg.ro, E.M. endre.mathe@agr.unideb.hu,V.T. turcus.violeta@uvvg.ro

3 National Institute for Economic Research "Costin C. Kiritescu" of the Romanian Academy/ Centre for Mountain Economy (CE-MONT), 725700 Suceava, Romania; V.B.B., Al.-S.N. sabin.nicula@ubbcluj.ro, V.T.

4 Doctoral School of Biomedical Sciences, University of Oradea, 410081 Oradea, Romania; A.A.N.

5 SC PlantExtrakt SRL, 407059 Rădaia, Cluj, Romania; N.K.O. neliolah@yahoo.com, F.R.P.F. flavia@plantextrakt.ro, R.F.B. ramona.burtescu@plantextrakt.ro

6 Faculty of Pharmacy, "Vasile Goldiș" Western University of Arad, 310414 Arad, Romania; N.K.O., E.C. chise.elisabeta@uvvg.ro

7 Centre for Research on Settlements and Urbanism, Faculty of Geography, Babeș-Bolyai University from Cluj-Napoca, 400006 Cluj-Napoca, Romania; Al.-S.N.

8 Department of Surgical Disciplines, Faculty of Medicine and Pharmacy, University of Oradea, 410081 Oradea, Romania; A.M.M. amaghiar@gmail.com

9 Institute of Nutrition, Faculty of Agricultural and Food Sciences and Environmental Management, University of Debrecen, H-4032 Debrecen, Hungary; E.M.

† These authors contributed equally to this work and are both considered first author.

* Correspondence: V. T. turcus.violeta@uvvg.ro

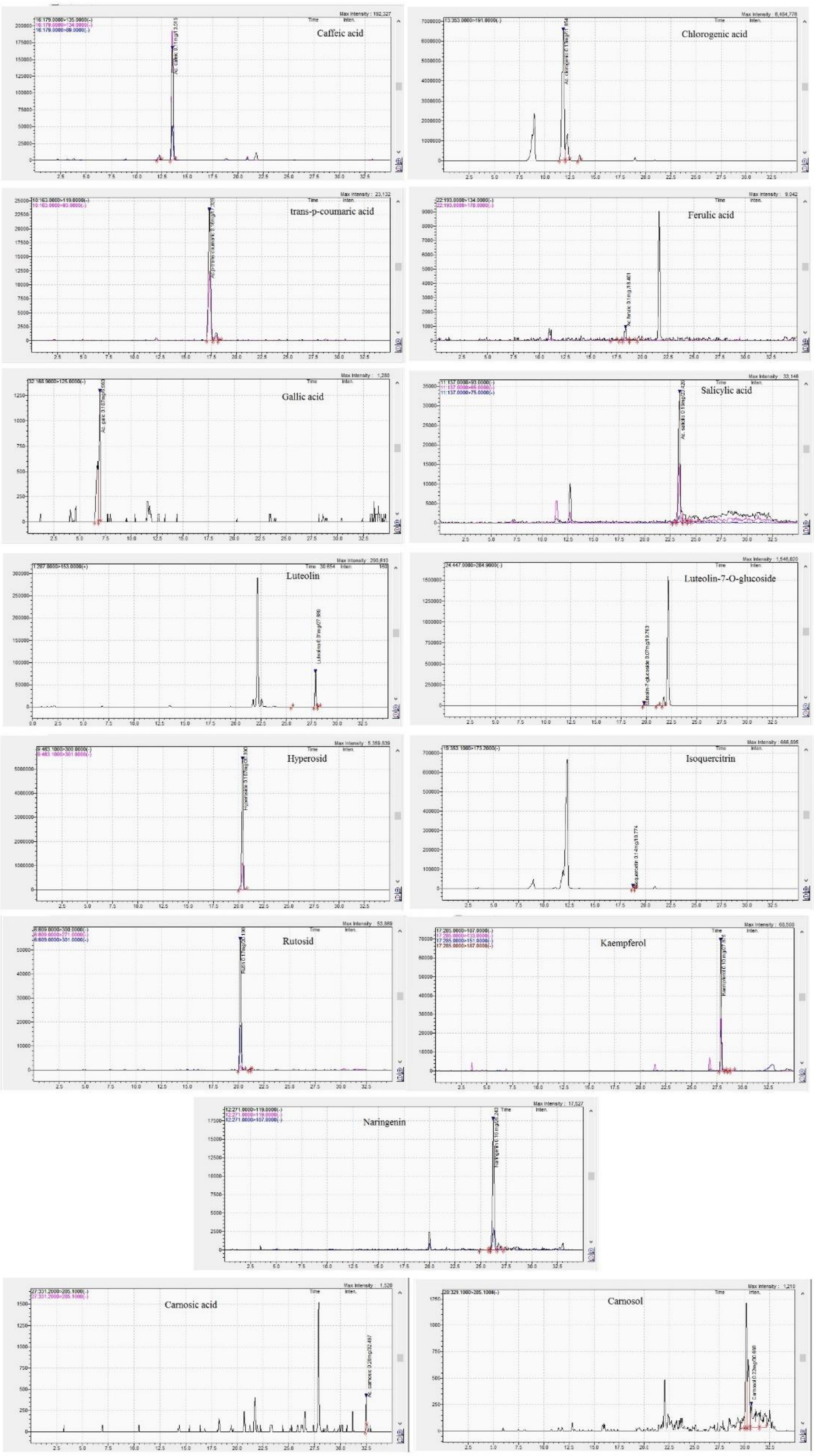

Figure S1. LC/MS chromatogram of the hydroalcoholic extract obtained from leaves of *T. farfara* L. from Rarău Mountains (RM). Compunds analysed

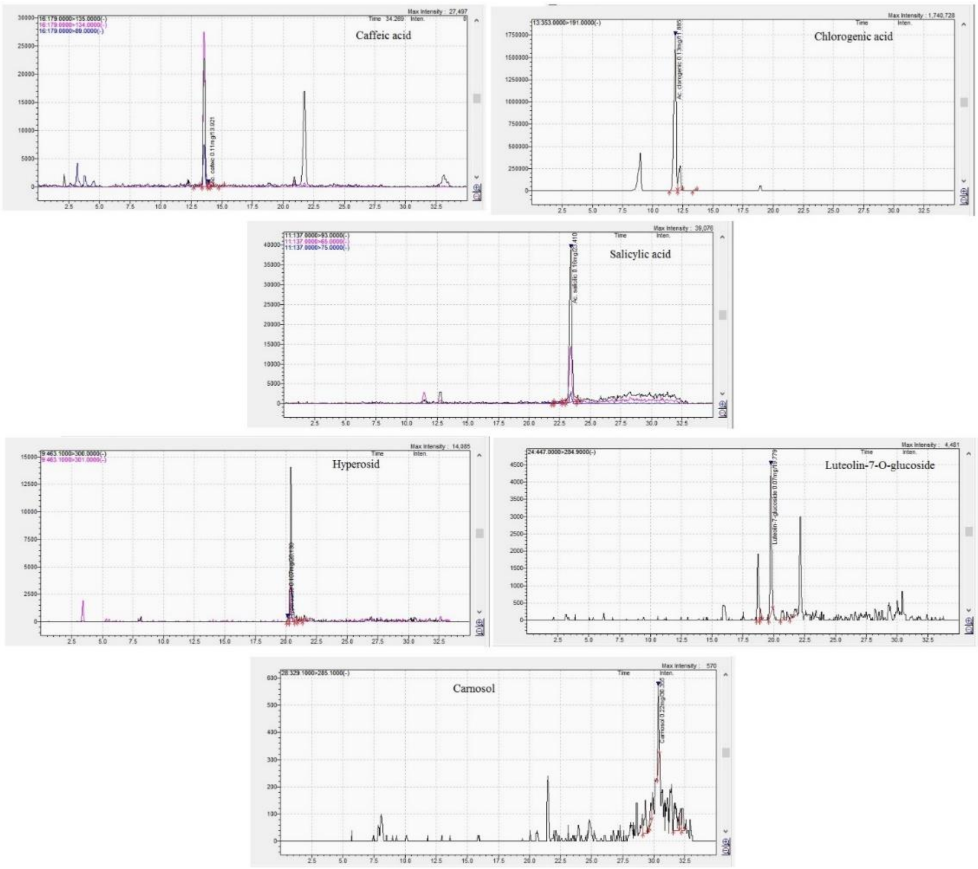

Figure S2. LC/MS chromatogram of the hydroalcoholic extract obtained from roots of *T. farfara* L. from Rarău Mountains (RM).

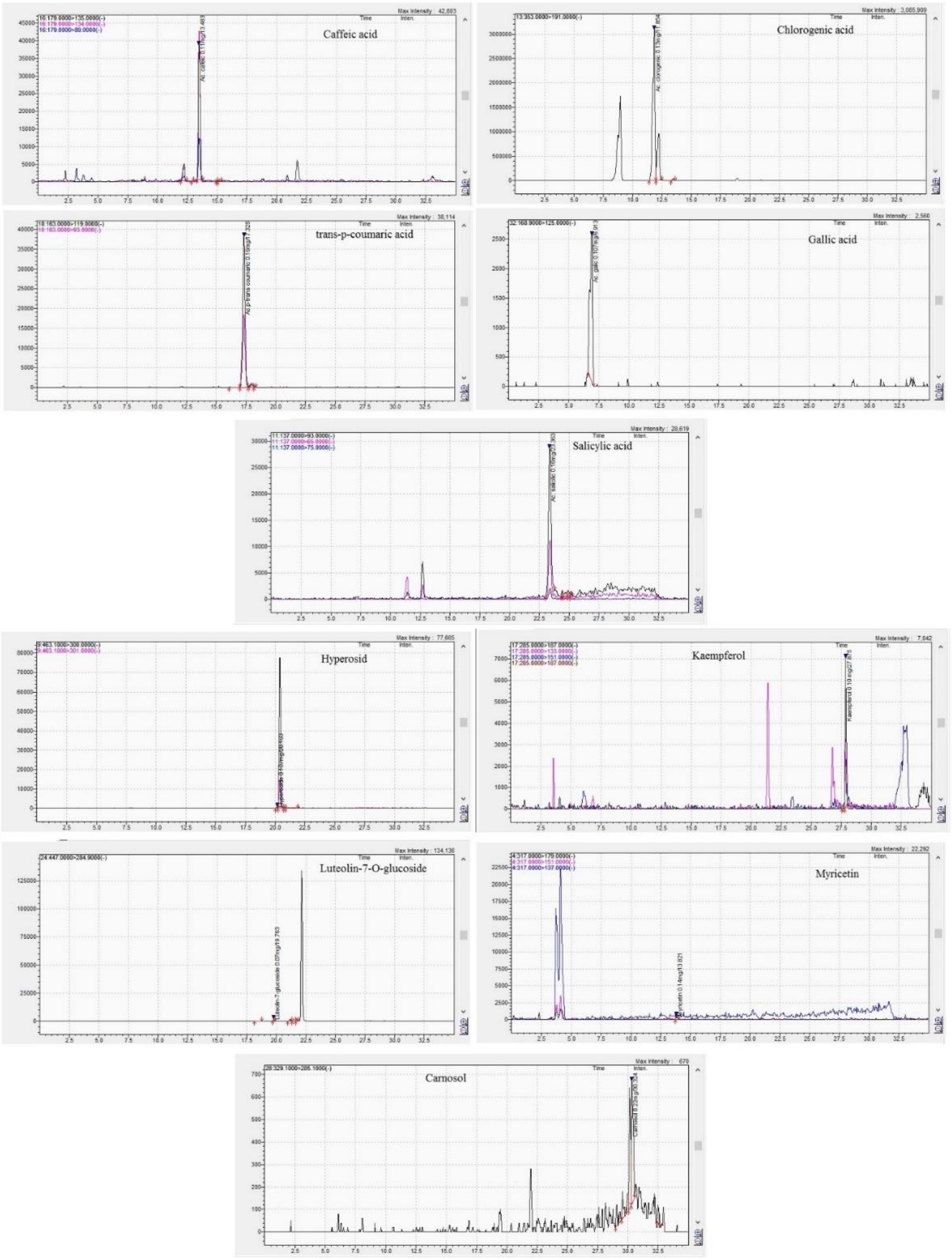

Figure S3. LC/MS chromatogram of the hydroalcoholic extract obtained from leaves of *T. farfara* L. from Vatra Dornei (VD).

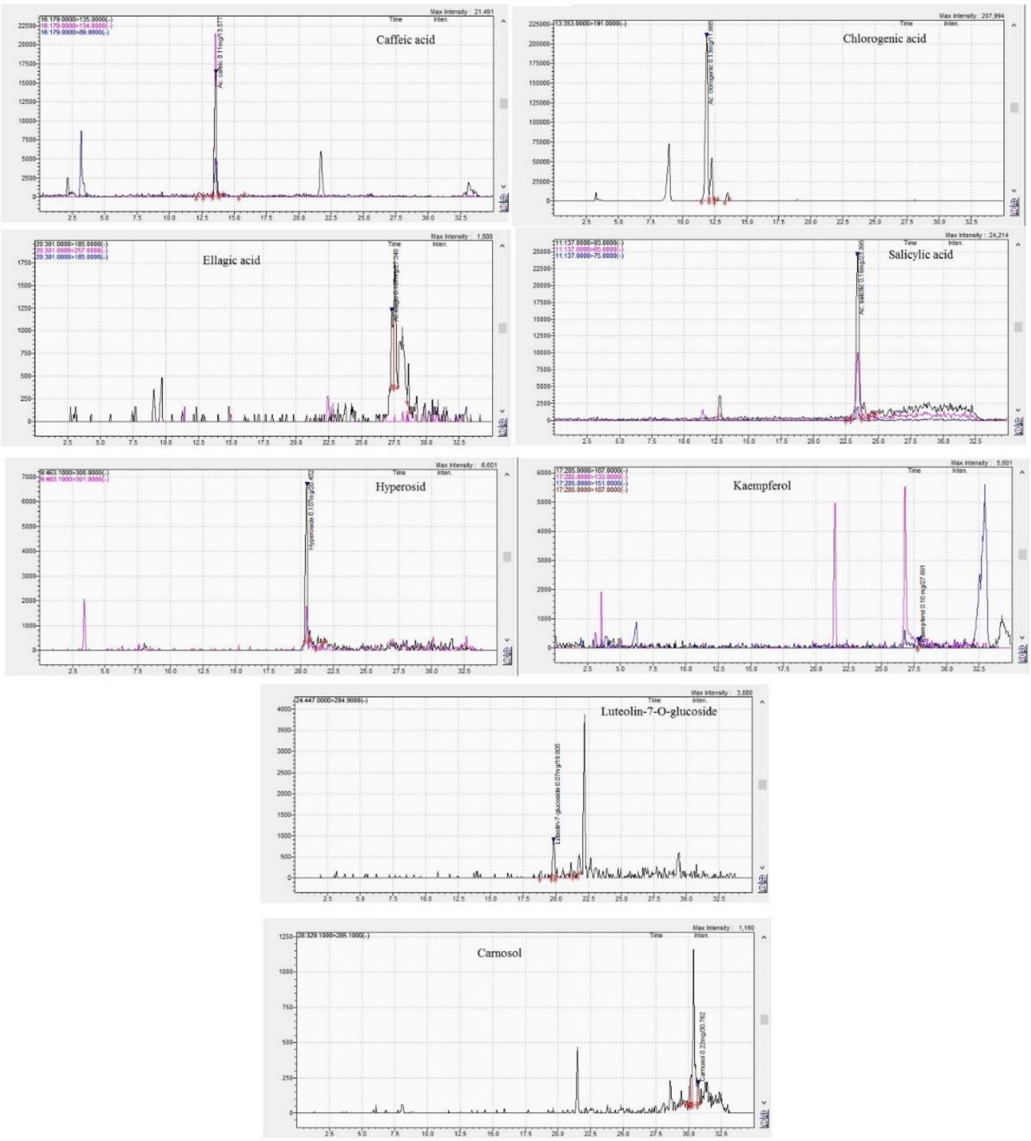

Figure S4. LC/MS chromatogram of the hydroalcoholic extract obtained from roots of *T. farfara* L. from Vatra Dornei (VD).

Table S1. Antioxidant activity of *T. farfara* extracts and isolated phytochemicals. Summarized results of original articles sorted by the plant part responsible and the extraction method.

Plant part	Preparation	Assay	Results	Ref.
Flower buds	Isolated indole alkaloids and lignans	2,2-Diphenyl-1-picrylhydrazyl (DPPH) assay	DPPH radical scavenging activity with IC_{50} values of 45.2 ± 2.9 and 29.2 ± 2.0 μM	[35]
	Ethyl acetate fraction	Lipid peroxidation, DPPH assay,	Inhibition of lipid peroxidation by Fe^{2+} and ascorbic acid in rat brain homogenates (IC_{50} = 6.3 mg/mL), DPPH scavenging activity 50 % at 14.3mg/mL	[36]
	Ethylacetate soluble fraction, isolated compounds	Nitroblue tetrazolium (NBT) assay	Ethyl acetate extract IC_{50} =1.8 μg/mL quercetin-3-*O*-β-L-arabinopyranoside - IC_{50} = 12.9 μM; quercetin-3-*O*-β-D-glucopyranoside - IC_{50} =35.6 μM	[37]

	Polusaccharides, water-soluble crude polysaccharides	Flow Injection Analysis paired with chemiluminescence (FIA-CL)	IC_{50} ascorbic acid < IC_{50} polysaccharides < IC_{50} crude polysaccharides	[38]
	Isolated compounds	Nitric oxide inhibition assay, lipopolysaccharide-treated RAW 264.7 cells	Nitric oxide inhibitory activity (3.5 μM ≤ IC_{50} ≤ 28.5 μM)	[39]
	Isolated compounds	Nitric oxide inhibition assay, lipopolysaccharide-treated RAW 264.7 cells	Dose-dependend nitric oxide inhibitory activity, from IC_{50} = 10.80 μM	[40]
	Isolated compound	Nitric oxide inhibition assay, lipopolysaccharide activated macrophages	Nitric oxide synthesis inhibition by bisabolene epoxide compound	[41]
	Isolated sesquiterpenoids	Lipopolysaccharide stimulated RAW 264.7 cells	Nitric oxide inhibitory activity	[42]
	Isolated sesquiterpenoids	*In vitro* and *in vivo* in PC12 cells and Oxidopamine (6-OHDA)-induced mouse model of Parkinson's disease	Protective effect against hydrogen peroxide and 6-hydroxydopamine-induced cytotoxicity, upregulated heme oxygenase 1 (HO-1) through interaction with Kelch-like ECH-associated protein 1 (Keap1)	[43]
	Isolated ECN sesquiterpenoid	*In vivo* study on partial sciatic nerve ligation induced peripheral neuropathic pain in albino mice	Prevention of suppresion of antioxidants such as glutathione, glutathione-S-transferase, catalase, superoxide dismutase, nuclear factor erythroid 2-related factor 2 (Nrf2), hemeoxygenase-1 and NAD(P)H-dependent quinone oxidoreductase	[44]
Leaves and flowers	Methanol extract	DPPH	Leaves antioxidant activty IC_{50} = 55 μg/mL	[14]
Leaves	Aqueous and hydro-ethanolic extracts	ABTS, DPPH	Aqueous [μmol Trolox equivalent/g dry weight]: 320.80 (ABTS), 51.63 (DPPH); hydro-ethanolic [μmol Trolox equivalent/g dry weight]: 390.00D±1.13 (ABTS), 268.52D±1.06 (DPPH)	[12]
	Ethyl acetate (EtOAc), methanol, water (MeOH)	ABTS, FRAP, CUPRAC, phosphomolybdenum, metal chelating assays	ABTS [mg Trolox equivalent/g dry weight]: EtOAc - 41.08; MeOH - 410.98; Water - 399.18	[45]
			FRAP [mg TE/g extract dry weight]: EtOAc - 49.98; MeOH - 465.31; Water - 380.25	
			CUPRAC [mg TE/g extract dry weight]: EtOAc - 93.78; MeOH - 677.09; Water - 53.38	
			Phosphomolybdenum [mg Trolox equivalent/g extract DW]: EtOAc - 1.54; MeOH - 2.26; Water - 1.44	
			Metal chelating [mg EDTA equivalent/g dry weight]: EtOAc - 13.11; MeOH - 6.16; Water - 4.98	
	Methanol extract	DPPH	EC_{50} = 14.54±0.72 μg/mL	[46]

	Ethanol extract	GC-MS with galvinoxyl free radical method	80-100% inhibition, highest radical scavenging efficiency out of 8 tested species	[47]
Leaves and roots	Acetone, methanol and ethanol extracts	DPPH, ABTS, FRAP	Leaves showed higher antioxidant activity than roots, and Lithuanian samples higher than French samples	[48]
Unspecified	Water and ethanol extracts	DPPH, yeast model	Water - 198.9 ± 0.71 μM ascorbate equivalent/g (DPPH); Ethanol -113.5 ± 0.00 μM ascorbate equivalent/g (DPPH); 20.9% inhibition of yeast oxidation by H_2O_2	[49]
	Methanol-water (8:2 v/v) extract	ABTS, FRAP	217.62 ± 5.35 μmol Trolox equvalent/g dry weight (TEAC), 455.64 ± 5.03 μmol Fe^{2+}/g dry weight (FRAP)	[50]

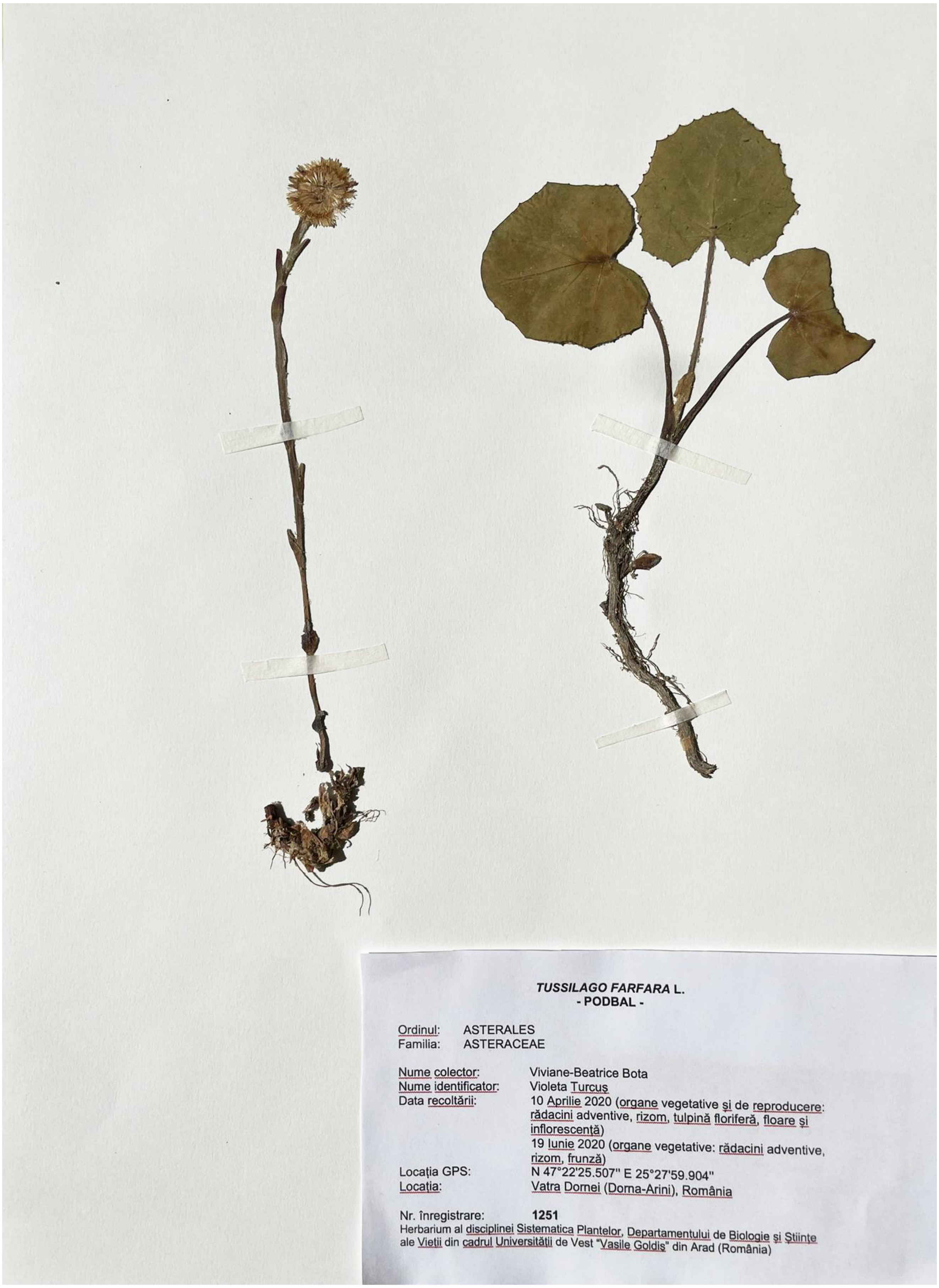

Figure S5. *Tussilago farfara* L. collected from Vatra Dornei. Registered with the voucher number 1251 in the Herbarium of the Plant Systematics discipline, part of the Department of Biology and Life Sciences of the "Vasile Goldiș" Western University of Arad (Romania).

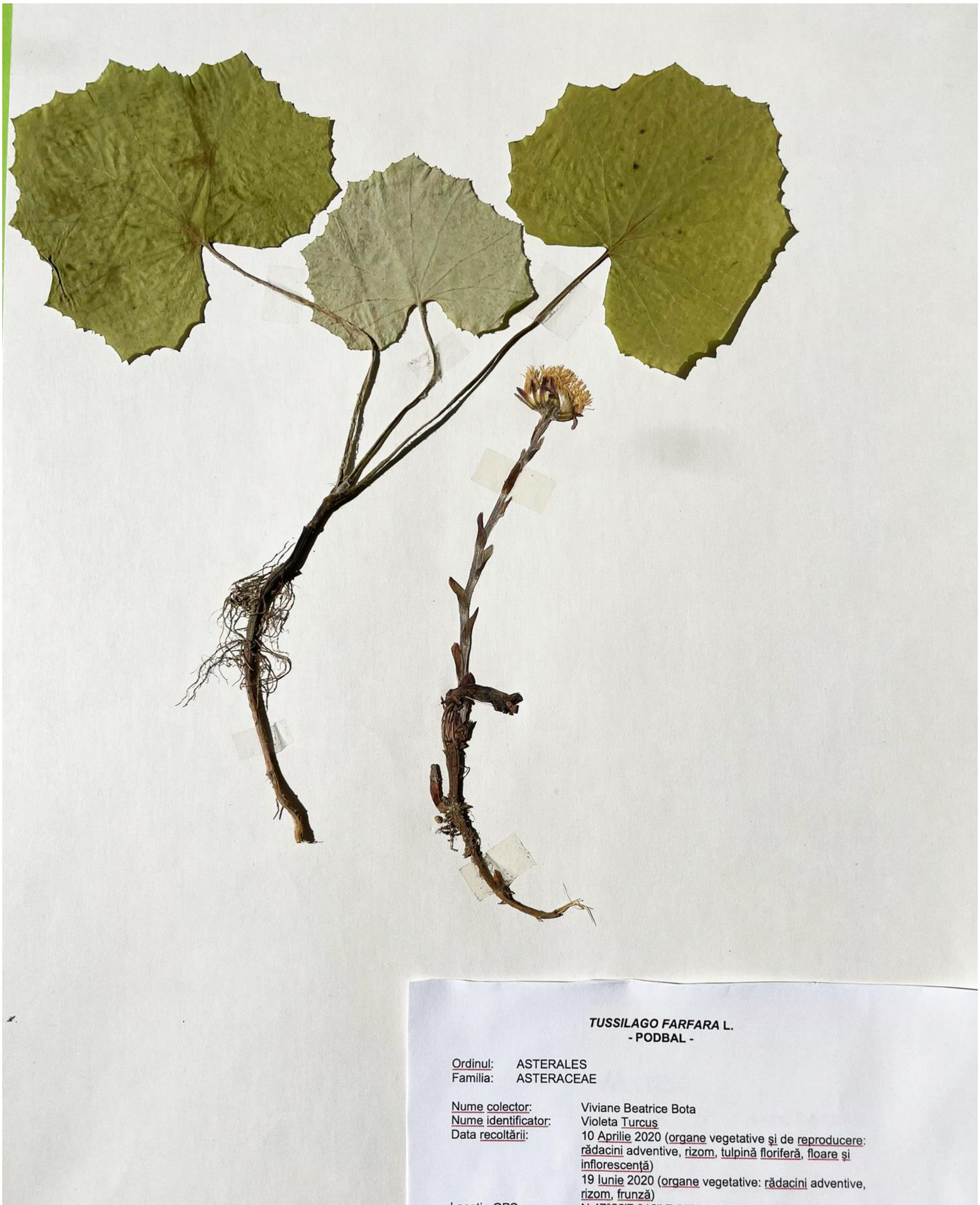

Figure S6. *Tussilago farfara* L. collected from Rarău Mountains. Registered with the voucher number 1252 in the Herbarium of the Plant Systematics discipline, part of the Department of Biology and Life Sciences of the "Vasile Goldiș" Western University of Arad (Romania).

Table S2. The standards for chromatographic and spectral data.

Standard	Retention time, min	m/z – main transition	MRM
**Caffeic acid**	13.8	179.0>135.0	Negative
***trans-p*-coumaric acid**	17.5	163.0>119.0	Negative
**Salicylic acid**	23.5	137.0>93.0	Negative
**Chlorogenic acid**	12.0	353.0>191.0	Negative
**Apigenin**	28.2	269.0>117.0	Negative
**Chrysin**	29.7	253.0>143.0	Negative
**Luteolin**	26.9	287.0>153.0	Positive
**Luteolin-*7-O*-glucoside**	19.9	447.0>284.9	Negative
**Quercetin**	25.7	300.9>151.0	Negative
**Rutoside**	20.3	609.0>300.0	Negative
**Naringenin**	26.3	271.0>119.0	Negative
**Carnosic acid**	32.0	331.2>285.1	Negative
**Ellagic acid**	27.2	301.0>185.0	Negative
**Carnosol**	30.7	329.1>285.1	Negative
**Kaempferol**	28.0	285.0>187.0	Negative
**Myricetin**	13.6	317.0>179.0	Negative
**Hyperoside**	20.3	463.1>300.0	Negative
**Isoquercitrin**	17.9	353.1>173.2	Negative
**Ferulic acid**	18.4	193.0>134.0	Negative
**Gallic acid**	7.0	168.9>125.0	Negative

Table S3. The standards used for LC/MS analyses.

Standard	Origin	Concentration range [mg/mL]	Calibration curve equation	Correlation factor	Detection limit [μg/mL]	Quantification limit [μg/mL]
**Caffeic acid**	Phytolab, Vestenbergsgreuth, Germany	0.11 – 1.10	Area = $4*10^7$*conc[mg/mL] - 319689	0.9998	3.20	4.80
***trans-p*-coumaric acid**	Phytolab, Vestenbergsgreuth, Germany	0.16 – 1.60	Area = $3*10^7$*conc[mg/mL] + 291065	0.9993	1.90	3.90
**Salicylic acid**	Merck, Darmstadt, Germany	0.16 – 1.60	Area = $4*10^7$*conc[mg/mL] + 44120	0.9997	1.50	2.00
**Chlorogenic acid**	Phytolab, Vestenbergsgreuth, Germany	0.13 – 1.30	Area = $2*10^8$*conc[mg/mL] - 269699	0.9997	5.00	8.00

**Apigenin**	Phytolab, Vestenbergsgreuth, Germany	0.10 – 0.98	Area = $2*10^8$*conc[mg/mL] + 15916	0.9999	0.20	0.30
**Chrysin**	Merck, Darmstadt, Germany	0.10 – 1.00	Area = $1*10^8$*conc[mg/mL] – 82818	0.9997	3.00	5.00
**Luteolin**	Phytolab, Vestenbergsgreuth, Germany	0.01 – 0.10	Area = $2*10^8$*conc[mg/mL] – 2295.4	0.9977	0.05	0.07
**Luteolin-*7-O*-glucoside**	Phytolab, Vestenbergsgreuth, Germany	0.07 – 0.70	Area = $1*10^9$*conc[mg/mL] – 700317	0.9990	3.00	4.00
**Quercetin**	Phytolab, Vestenbergsgreuth, Germany	0.09 – 0.91	Area = $5*10^7$*conc[mg/mL] – 9556	0.9964	0.80	1.10
**Rutoside**	Phytolab, Vestenbergsgreuth, Germany	0.17 – 1.70	Area = $2*10^8$*conc[mg/mL] – 191937	0.9996	4.00	6.00
**Naringenin**	Phytolab, Vestenbergsgreuth, Germany	0.16 – 1.60	Area = $3*10^8$*conc[mg/mL] – 43443	0.9999	0.60	0.90
**Carnosic acid**	Phytolab, Vestenbergsgreuth, Germany	0.28 – 2.80	Area = 10^7*conc[mg/mL] – 99360	0.9994	4.00	6.00
**Ellagic acid**	Phytolab, Vestenbergsgreuth, Germany	0.107 – 1.070	Area = 14987*conc[mg/mL] – 138.52	0.9982	3.70	5.50
**Carnosol**	Phytolab, Vestenbergsgreuth, Germany	0.022-0.220	Area = 10^9*conc[mg/mL] – 253279	0.9997	1.00	2.00
**Kaempferol**	Phytolab, Vestenbergsgreuth, Germany	0.10 – 1.00	Area = 10^7*conc[mg/mL] – 20574	0.9996	0.80	1.20
**Myricetin**	Phytolab, Vestenbergsgreuth, Germany	0.140 – 1.400	Area = 26499*conc[mg/mL] – 41.803	0.9997	0.60	0.90
**Hyperoside**	Phytolab, Vestenbergsgreuth, Germany	0.012 – 0.107	Area = $4*10^8$*conc[mg/mL] – 567182	0.9986	0.60	0.90
**Isoquercitrin**	Phytolab, Vestenbergsgreuth, Germany	0.140 – 1.400	Area = 4727*conc[mg/mL] + 68.172	0.9973	2.90	5.80

**Ferulic acid**	Phytolab, Vestenbergsgreuth, Germany	0.100-1.000	Area = $5*10^6$*conc[mg/mL] – 50000	0.9992	4.00	6.00
**Gallic acid**	Phytolab, Vestenbergsgreuth, Germany	0.107-1.070	Area = $8*10^6$*conc[mg/mL] – 37131	0.9999	1.90	2.80

Table S4. The spectral calibration curves data.

	**Calibration curve equation**	**Correlation factor**
**Total flavonoids expressed in rutoside**	Absorption = 0.024*conc[μg/mL] + 0.0031	0.9970
**Total phenolic acids expressed in caffeic acid**	Absorption = 0.0581*conc[μg/mL] + 0.1473	0.9855
**Trolox / FRAP**	Absorption = 6.0343 *conc[μM/g] + 0.0186	0.9920
**Trolox / CUPRAC**	Absorption = 3.7061*conc[μM/g] + 0.0093	0.9738
